# Evidence-Based Interventions to Reduce the Incidence of Common Multidrug-Resistant Gram-Negative Bacteria in an Adult Intensive Care Unit

**DOI:** 10.7759/cureus.39979

**Published:** 2023-06-05

**Authors:** Abdulhakeem Althaqafi, Muhammad Yaseen, Fayssal Farahat, Adeeb Munshi, Fahad M Al-Hameed, Majid M Alshamrani, Asim Alsaedi, Abdulfattah Al-Amri, Hafizah Chenia, Sabiha Y Essack

**Affiliations:** 1 College of Medicine, King Saud bin Abdulaziz University for Health Sciences, Jeddah, SAU; 2 Infectious Diseases, King Abdullah International Medical Research Center, Jeddah, SAU; 3 Medicine/Infectious Diseases, King Abdulaziz Medical City, Jeddah, SAU; 4 Infection Prevention and Control, Bradford Teaching Hospitals National Health Service (NHS) Foundation Trust, Bradford, GBR; 5 Public Health and Community Medicine, King Saud bin Abdulaziz University for Health Sciences, Riyadh, SAU; 6 Public Health and Community Medicine, Menoufia University, Shibin El Kom, SAU; 7 Infection Prevention and Control, King Abdulaziz Medical City, Riyadh, SAU; 8 Intensive Care Unit, King Abdullah International Medical Research Center, Jeddah, SAU; 9 Intensive Care Unit, King Abdulaziz Medical City, Jeddah, SAU; 10 College of Medicine, King Saud bin Abdulaziz University for Health Sciences, Riyadh, SAU; 11 Infection Prevention and Control, King Abdullah International Medical Research Center, Riyadh, SAU; 12 Infection Prevention and Control, King Abdullah International Medical Research Center, Jeddah, SAU; 13 Infection Prevention and Control, King Abdulaziz Medical City, Jeddah, SAU; 14 Microbiology, King Abdullah International Medical Research Center, Jeddah, SAU; 15 Microbiology, King Abdulaziz Medical City, Jeddah, SAU; 16 Microbiology, Discipline of Microbiology, School of Life Sciences, College of Agriculture, Engineering and Sciences, University of Kwazulu-Natal, Durban, ZAF; 17 Antimicrobial Research Unit, University of Kwazulu-Natal, Durban, ZAF

**Keywords:** saudi arabia, intensive care unit, interventions, incidence, multidrug-resistant, gram-negative bacteria

## Abstract

Background

Multidrug-resistant Gram-negative bacteria (MDR-GNB) present a significant and escalating hazard to healthcare globally. Context-specific interventions have been implemented for the prevention and control of MDR-GNB in several healthcare facilities. The objective of this study was to implement and evaluate the effectiveness of evidence-based interventions in the incidence and dissemination of MDR-GNB.

Methods

This was a pre-and post-intervention study conducted in three phases at King Abdulaziz Medical City Jeddah, Saudi Arabia. During Phase-1, the data on each of the four MDR-GNB (*Acinetobacter baumannii, Klebsiella pneumoniae, Pseudomonas aeruginosa, and Escherichia coli*) were collected prospectively. Genomic fingerprinting was performed on isolates using enterobacterial repetitive intergenic consensus-polymerase chain reaction (ERIC-PCR) to determine clonality and establish a link between different strains within and between the hospital wards/units. In the second phase, targeted interventions were implemented in the adult intensive care unit (ICU) based on previously determined risk factors and included the education of healthcare workers on hand hygiene, disinfection of patients’ surrounding, daily chlorhexidine baths, and disinfection rooms on discharge with hydrogen peroxide fogging after MDR-GNB patients were discharged. An antibiotic restriction protocol was simultaneously implemented as part of the hospital antibiotic stewardship program. In the third phase, the effectiveness of the interventions was evaluated by comparing the incidence rate and clonality (using ERIC-PCR genetic fingerprints) of MDR-GNB before and after the intervention.

Results

A significant reduction of MDR-GNB was observed in Phase-2 and Phase-3 compared with Phase-1. The mean incidence rate of MDR-GNB per 1000 patient days in Phase-1 (pre-intervention) was 11.08/1000, followed by 6.07 and 3.54/1000 in Phase-2 and Phase-3, respectively. A statistically significant reduction was observed in the incidence rate of MDR-GNB in the adult ICU (P=0.007), whereas no statistically significant decrease (P=0.419) was observed in areas other than the adult ICU. Two *A. baumannii* strains appear to be circulating within the ICU environment with reduced frequency in Phase-2 and Phase-3 compared to Phase-1.

Conclusion

There was a significant reduction in the incidence of MDR-GNB in the adult ICU due to the successful implementation of both infection control and stewardship interventions, albeit challenging to ascertain the relative contribution of each.

## Introduction

Antibiotic resistance is among the leading global health problems facing modern medicine [1]. Resistant bacteria are frequently found in intensive care units (ICUs) due to the widespread use of broad-spectrum antimicrobials and invasive devices among these patients compared with other wards in healthcare facilities [2]. Gram-negative bacteria (GNB) are prominent nosocomial isolates, specifically *Acinetobacter baumannii (A. *baumannii)*, Klebsiella pneumoniae (K. *pneumoniae)*, Pseudomonas aeruginosa (P. *aerugino*sa), and Escherichia coli (E. Coli) *[3]. Multidrug-resistant (MDR) GNB infections have become considerably challenging during the last two decades, especially in developing countries, and are linked with elevated morbidity and mortality, including prolonged hospital stay [4,5].

Several risk factors could be associated with an increased incidence of MDR-GNB, including antibiotic overuse and misuse, lack of compliance to and/or sub-optimal infection prevention and control (IPC) principles, unskilled healthcare workers, an unhygienic environment that facilitate the spread of resistant organisms and deficient antibiotic use, and resistance surveillance systems [6]. Antimicrobial stewardship intervention has been shown to be effective in improving the appropriateness of carbapenem use and combating antimicrobial resistance [7].

Despite the inclusion of IPC and antibiotic stewardship in the Saudi Arabian strategic plan to mitigate the antibiotic resistance challenge [8], there is little information on the implementation and outcome of IPC interventions to date, particularly in healthcare facilities where elevated resistance has become endemic [9]. Knowledge of the local epidemiology of antibiotic-resistant isolates in ICU-acquired MDR-GNB could inform IPC and/or antimicrobial stewardship programs to achieve better patient outcomes [10].

The study's objective was to assess the impact of evidence-based interventions on the incidence and dissemination of common MDR-GNB in an adult ICU at King Abdulaziz Medical City, Jeddah, Saudi Arabia.

## Materials and methods

Ethical approval 

The study was approved by the Institutional Review Board (IRB) at King Abdulaziz Medical City in Saudi Arabia (RJ13/032/J) and the Biomedical Research Ethics Committee (BREC) at the University of Kwazulu-Natal (BE279/13). All data were anonymously collected and interpreted. Additionally, all patients signed a general consent form at the time of hospital admission as part of the hospital policy.

Study design and setting 

The study was conducted at King Abdulaziz Medical City Jeddah (KAMC-JD) in Saudi Arabia. KAMC-JD is an over 500-bed tertiary care hospital serving the National Guards and their eligible dependents in the Western Region of Saudi Arabia. Four adult ICUs house combined medical and surgical patients with a capacity of 28 beds. This was a pre-and post-intervention study conducted over 26 months (January 2015 to February 2017) evaluating the impact of evidence-based interventions on the incidence of MDR-GNB infections. The study was divided into three phases: Phase-1 (pre-intervention), Phase-2 (intervention), and Phase-3 (post-intervention).

Data collection

In Phase-1 (January 2015-June 2016), the data on all isolates of each of the four MDR-GNB were collected prospectively. The organisms included *A. baumannii, K. pneumoniae, P. aeruginosa, and E. coli.* The bacterial isolates were collected prospectively from routine clinical samples taken from patients presenting with bloodstream, respiratory tract, skin, soft tissues, and urinary tract infections. Only isolates with healthcare-associated infection were included in the study using the standard definition of infection occurring 48 hours after admission. Each organism was included in the study only once, at the time of the first positive result. The definition of the MDR by the experts from the European Centre for Disease Prevention and Control (ECDC) and the Centers for Disease Control and Prevention (CDC) was used for this study, where MDR was defined as the organism to be non-susceptible to at least one antibiotic agent in three or more classes of antibiotics [11]. Isolates were categorized as MDR or susceptible on the basis of their minimum inhibitory concentrations determined on the automated Vitek® 2 System (BioMérieux, Marcy-l'Étoile, France) and interpreted according to CLSI guidelines. The incidence rate of those bacteria was calculated using patient-days statistics extracted from the hospital's electronic records.

In Phase-2 (July-October 2016), targeted interventions were implemented in adult ICUs. The period for the development and implementation of interventions was four months. The interventions included daily bathing of all patients with chlorhexidine solution, cleaning and disinfection of patient’s surrounding twice a day at each change of nursing shift, education of healthcare workers on hand hygiene (HH) compliance, and terminal cleaning and disinfection of rooms occupied by MDR-GNB patients with hydrogen peroxide solution using the fogging technique. An antibiotic restriction form to reduce and control the consumption of restricted antibiotics, especially carbapenems, was simultaneously implemented as part of the hospital antimicrobial stewardship program. The data collection on bacterial isolates continued in Phase-2 also. 

The data collection continued in Phase-3 (November 2016-Februry 2017) to evaluate the effectiveness of the interventions by comparing the incidence rate of MDR-GNB before and after the intervention. The interventions started in phase two and continued throughout phases two and three. HH observation was also carried out again in phase three to determine the improvement in HH compliance as a result of staff education.

Data sources

The data for all phases were collected using multiple data sources including

• Microbiology laboratory records on Gram-negative bacteria and their sensitivity profiles. This information was available electronically using the hospital information system. 

• Patients' admission information sent to the infection control office by Information Services Department (ISD) daily was used for calculating hospital patient days that served as the denominator for determining the incidence rate of hospital-acquired infections.

• Patients' charts, as well as the electronic patients' record, were used to collect demographic and clinical information and identify risk factors, i.e. antibiotic therapy and use of invasive devices or procedures. Patients' non-electronic charts were also reviewed for any missing information in the electronic records because the electronic system was recently implemented in our institution.

HH observation

HH observational audits were conducted in the clinical units/wards to determine HH compliance by healthcare workers. Direct HH monitoring was carried out using HH methodology and monitoring tools developed by the World Health Organization (WHO) [12]. The infection control practitioners carried out direct observation of practices in clinical areas. The services included medicine, surgery, oncology, adult ICUs, pediatric and neonatal ICUs, and a hemodialysis unit. A minimum of 200 HH opportunities were collected from each service. The HH results were categorized by clinical services, professional category (physicians, nurses, and other allied health workers), and five moments/indications as per the WHO guidelines. HH observations were carried out in Phase-1 (pre-intervention) and Phase-3 (post-intervention) to evaluate the effectiveness of educational sessions for healthcare workers.

Enterobacterial repetitive intergenic consensus-polymerase chain reaction (ERIC-PCR)

In Phase-1, genomic DNA extraction was carried out for 148 samples using the Thermo Scientific® GeneJet Genomic DNA purification kit (Thermo Fisher Scientific, Johannesburg, South Africa), following the manufacturer’s instructions. Isolates underwent genomic fingerprinting using ERIC-PCR to establish the clonality and the link of different strains within and between the hospital wards/units. The primers for the ERIC-PCR included ERIC 1 5’-ATG TAA GCT CCT GGG GAT TCA C-3’ and ERIC2 5’-AAG TAA GTG ACT GGG GTG AGC G-3’ [13].

For ERIC-PCR, the total reaction size was 10 µL, which contained 2 µL of template DNA, 0.1 µL of each primer (100 μM), 2.8 µl nuclease-free water, and 5 µL of DreamTaq Green Polymerase Master Mix 2× (Thermo Fisher Scientific). PCR reactions were conducted in an Applied Biosystems 2720 programmable thermal cycler (Thermo Fisher Scientific, South Africa) with the following PCR conditions: an initial denaturation step of 94 °C for 3 min, 30 cycles of 30 s of denaturation at 94 °C, 1 min of annealing at 50 °C, 8 min of extension at 65 °C, and a final elongation step of 16 min at 65 °C, followed by storage at 4 °C.ERIC-PCR products were loaded into 1% (w/vol) agarose gels, together with Quick Load® 1-kb DNA ladder (New England Biolabs, USA) and subjected to electrophoresis at 100V for 2 h. Gels were stained in a solution containing 0.1 mg/ml ethidium bromide for 15 min. Amplification products were visualized by UV trans-illumination, and images were captured using a gel documentation system (Syngene, UK) and recorded for further analyses.

ERIC profiles were analyzed using Bionumerics software (version 7.6, Applied Maths, TX, USA). All DNA fragment sizes within each gel were normalized using the Quick Load® 1-kb DNA molecular weight marker as the external reference standard. The similarity between each strain was determined from the homology matrix using the Dice coefficient. Dendrograms were constructed based on the averaged similarity of the matrix using the Jacquard coefficient and the Unweighted Pair-Group Method (UPGMA) algorithm with optimization and band tolerance set a 1% (version 7.6, Applied Maths, TX, USA). The co-phenetic correlation value was calculated for the dendrogram to measure generated clusters' reliability. Clusters were defined based on a similarity cut-off of 80%.

ERIC-PCR genomic fingerprinting for MDRA. *A. Baumannii* isolates was also carried out in Phase-3 to evaluate whether the remaining strains were similar to those in Phase 1 and the variation, if any, after interventions. 

Data analysis

The data were analyzed using IBM SPSS Statistics for Windows, Version 20 (Released 2011; IBM Corp., Armonk, New York, United States). One-way analysis of variance (ANOVA) was carried out to determine the difference between different phases of the study. The level of significance was determined at p-value < 0.05.

## Results

MDR-GNB incidence 

**Figure 1:**
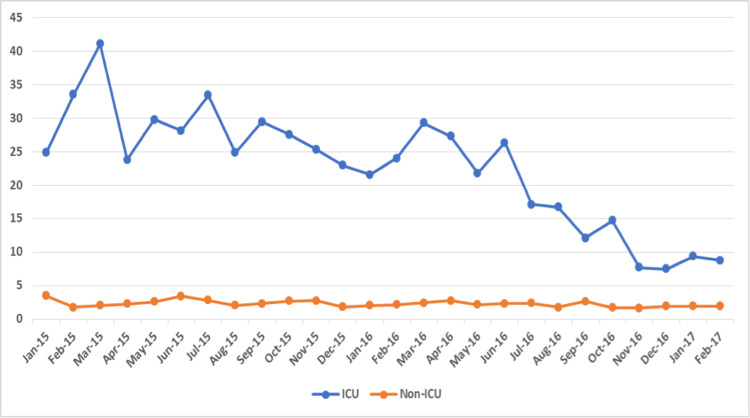
Incidence rate of MDR-GNB per 1000 patient days. MDR-GNB: Multidrug-resistant Gram-negative bacteria; ICU: intensive care unit

A total of 1090 MDR-GNB isolates were collected throughout the hospital over 26 months. The number of isolates collected from the adult ICU was 456 (42%), and the incidence rate was much higher in the adult ICU (22.8/1000 patient-days) compared to the rest of the hospital combined areas (2.3/1000 patient-days). The rate of MDR-GNB was higher in adult ICUs throughout the study period, as shown in Figure 1.

The mean incidence of MDR-GNB in the adult ICU in Phases 1, 2, and 3 was 11.08, 6.07, and 3.54 per 1000 patient days. The results show significant differences among the three phases regarding the incidence in adult ICU as indicated by the p-value of the F test (p=0.0005). The mean incidence of MDR-GNB inwards/units other than adult ICU (without interventions) in Phases 1, 2, and 3 was 0.97, 0.85, and 0.72 per 1000 patient days, as shown in Table 1.

**Table 1:** Mean incidence rate of MDR-GNB per 1000 patient days by phases. *A. baumannii: Acinetobacter baumannii; E. coli: Escherichia coli; K. pneumoniae: Klebsiella pneumoniae; P. aeruginosa: Pseudomonas aeruginosa; *ICU: intensive care unit; MDR-GNB: multidrug-resistant Gram-negative bacteria

	ADULT ICU				Other Wards/Units			
	Phase-1	Phase-2	Phase-3	P value	Phase-1	Phase-2	Phase-3	P value
Overall MDR-GNB	11.08	6.07	3.54	0.0005	0.97	0.85	0.72	0.372
A. baumannii	12.67	7.21	2.81	0.0001	0.64	0.65	0.57	0.879
E. coli	1.33	0.29	0	0.03	0.46	0.29	0.21	0.007
K. pneumoniae	9.46	4.49	3.51	0.0001	0.82	0.68	0.55	0.242
P. aeruginosa	4.4	3.17	2.53	0.115	0.48	0.5	0.36	0.419

The results show insignificant differences among the three phases, as indicated by the p-value (p=0. 314).

For individual MDR-GNB, the mean incidence per 1000 patient days of* A. baumannii* in Phases 1, 2, and 3 in the adult ICU was 12.67, 7.21, and 2.81, respectively. There was an overall reduction of 78%, and the results were found to be statistically significant (p = 0.0001). Likewise, the mean incidence of *E. coli* in Phases 1, 2, and 3 was 1.33, 0.29, and 0.00, respectively (p = 0.030). The mean incidence of *E. coli* in areas other than adult ICU was also reduced post-intervention (p = 0.007). The number of *E. coli* was low in all phases, so the reduction may have been coincidental, as no interventions were implemented in those areas. The mean incidence of *K. pneumoniae* in Phases 1, 2, and 3 was 9.46, 4.49, and 3.51, respectively. There was an overall reduction of 62%, and the results were statistically significant (p = 0.0001). Similarly, the mean incidence of *P. aeruginosa *in Phases 1, 2, and 3 was 4.40, 3.17, and 2.53, respectively. Despite some reduction in the rate, the results were not statistically significant (p = 0.115). 

HH compliance

HH compliance was monitored in six clinical areas in Phase-1. The HH compliance in all six areas was 87%, 90%, 92%, 75%, 95%, and 89% in the medicine, surgery, oncology, adult ICUs, pediatric and neonatal ICUs, and hemodialysis units, respectively. The lowest compliance was observed in the adult ICU with only 75%. The HH compliance in the post-intervention phase increased to 88%, which is statistically significant. In Phase-3, HH has only been monitored in adult ICUs. The interventions, including HH education, were implemented in the adult ICU. That is the reason post-intervention monitoring was done only in adult ICUs.

ERIC-PCR DNA genomic fingerprinting

A total of 148 isolates were tested in Phase-1. The isolates included *A. baumannii* (n = 55), K. pneumoniae (n = 48), *P. aeruginosa* (n = 26), and *E. coli* (n = 19). Fingerprints were examined for differences and similarities in the number and sizes of PCR amplicons. ERIC-PCR results of *A. baumannii* pre- and post-intervention are presented as an illustrative example.

**Figure 2:**
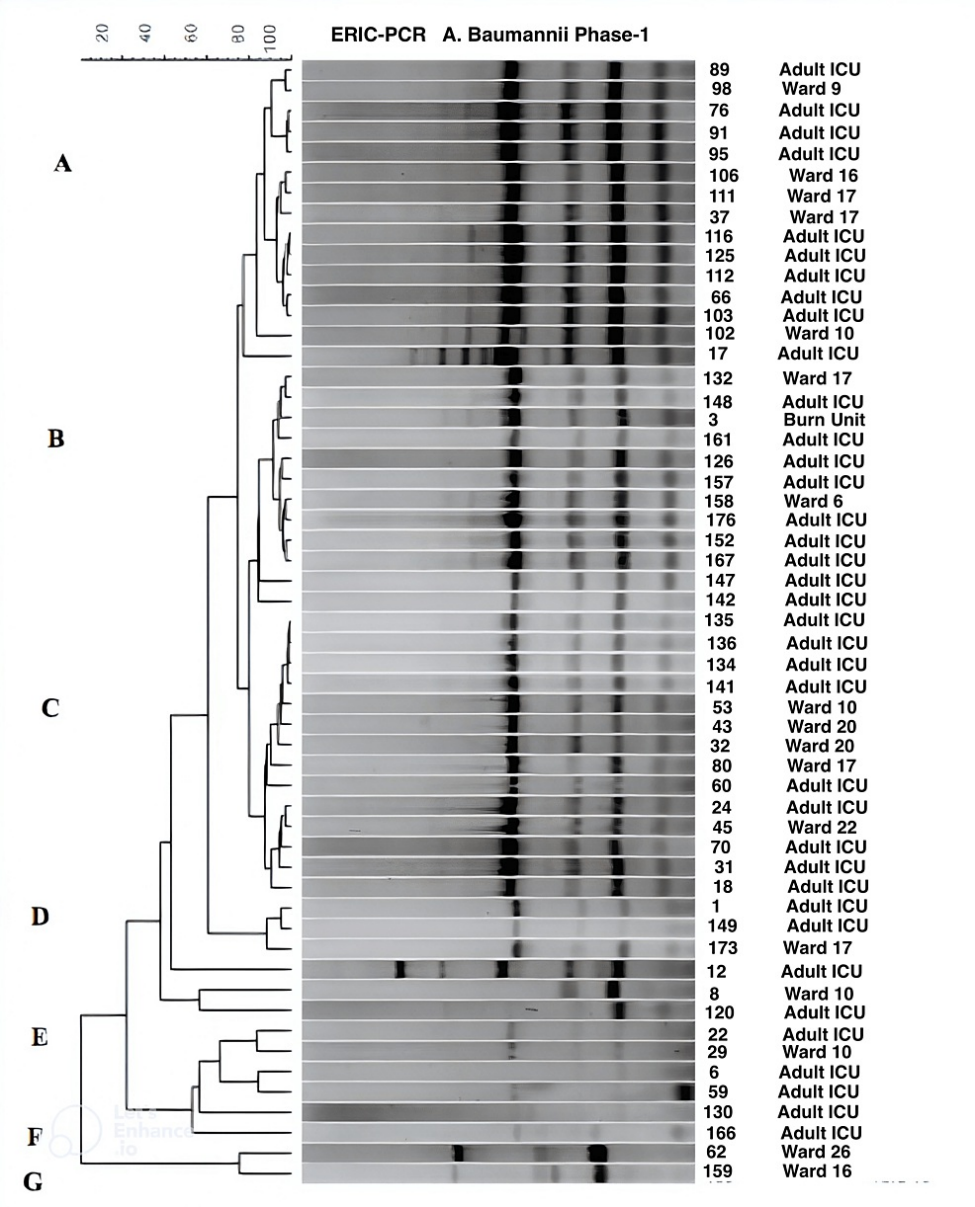
Dendrogram showing the cluster analysis based on ERIC-PCR fingerprinting patterns using the Jacquard index and UPGMA clustering for A. baumannii isolates collected during Phase-1 (pre-intervention) from different hospital wards/units. The scale at the top represents percentage similarity. ICU: Intensive care unit; ERIC-PCR: enterobacterial repetitive intergenic consensus-polymerase chain reaction; A. baumannii: Acinetobacter baumannii; UPGMA: unweighted pair-group method

Three major clusters (A-C) and four smaller clusters were observed with the *A. baumannii* isolates, the majority of which had very similar banding patterns (Figure 2), suggesting the spread of two to three clonal strains.

Isolates within all three major clusters demonstrated similarity with four primary bands and differed with respect to fainter secondary and tertiary amplicons. The majority of the isolates showing a high degree of banding similarity were from the adult ICU indicating the possible transmission of bacteria within the unit and then transfer to patient wards following departure from ICU. Isolates within clusters D-F demonstrated less similarity to the three major clusters, with some of these non-clonal isolates from the adult ICU being transferred to other wards. Cluster G isolates did not appear to originate in the adult ICU. 

**Figure 3:**
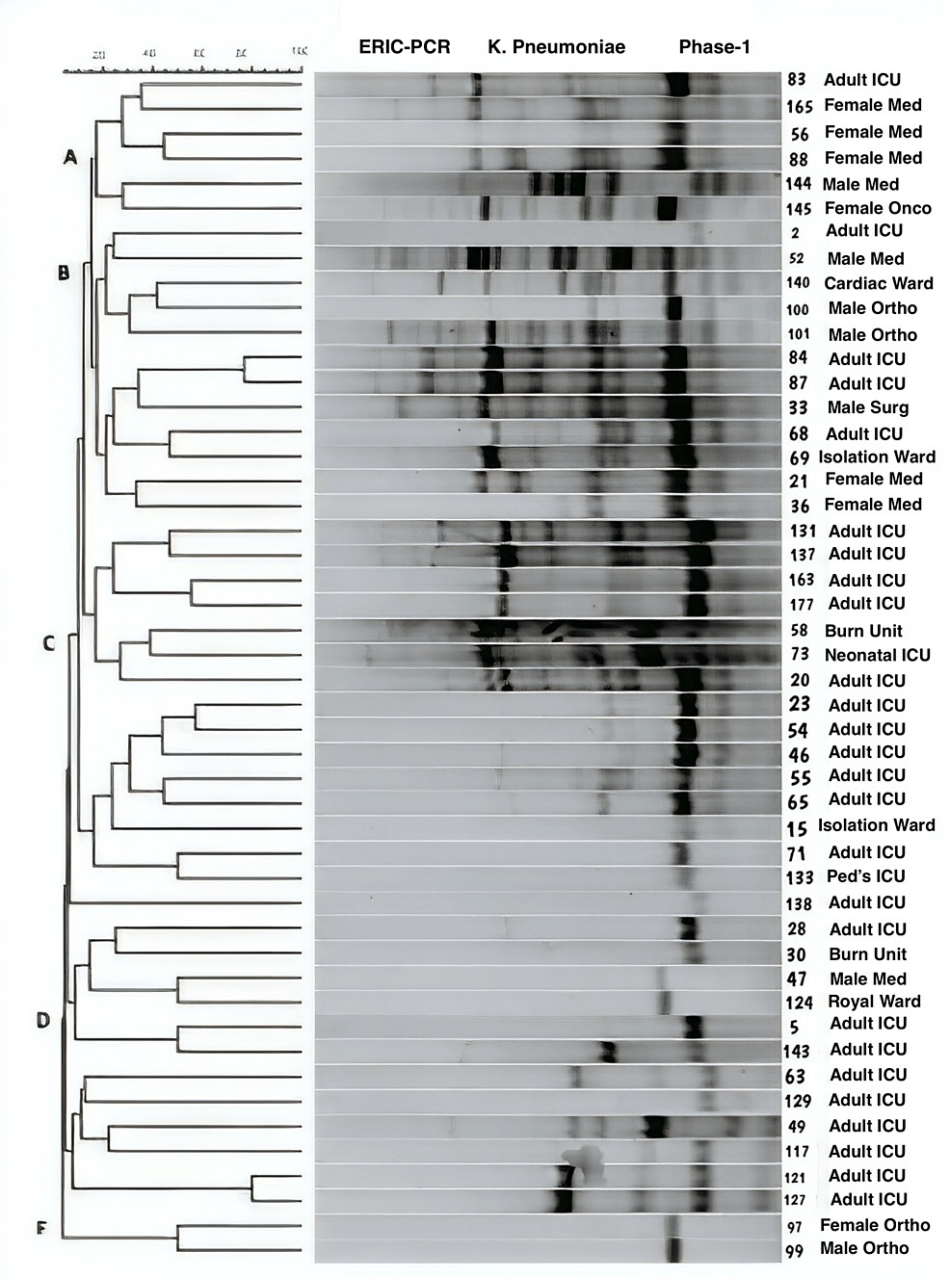
Dendrogram showing the cluster analysis based on ERIC-PCR fingerprinting patterns using the Jacquard index and UPGMA clustering for K. pneumoniae isolates collected during Phase-1 (pre-intervention) from different hospital wards/units. The scale at the top represents percentage similarity. K. pneumoniae: Klebsiella pneumoniae; ICU: intensive care unit; Med: medical; Onco: oncology; Ortho: orthopedics; Surg: surgery; Ped's: pediatrics; ERIC-PCR: enterobacterial repetitive intergenic consensus-polymerase chain reaction; UPGMA: unweighted pair-group method

More diversity was observed with the *K. pneumoniae* isolates, which demonstrated varied ERIC-PCR fingerprints (Figure 3).

**Figure 4:**
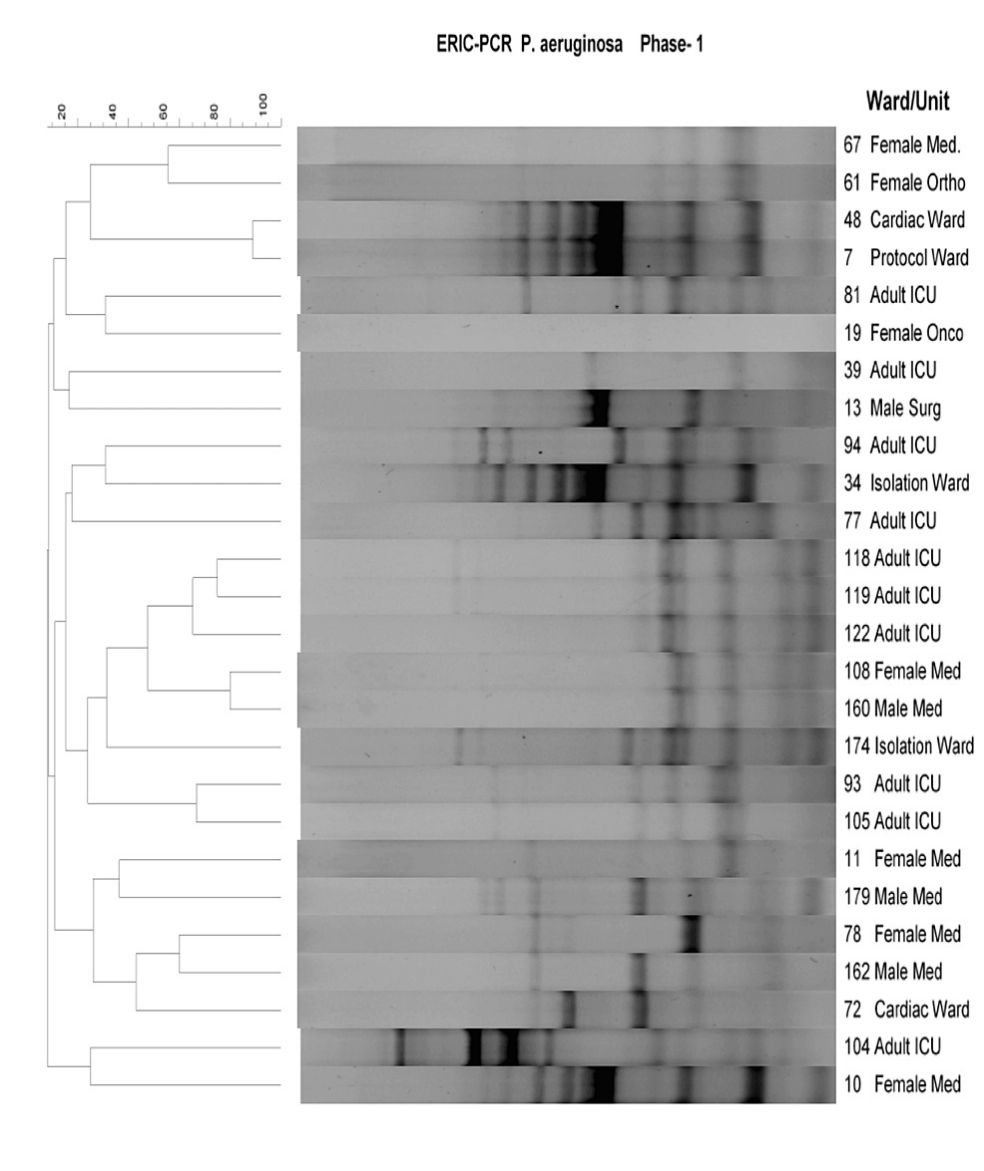
Dendrogram showing the cluster analysis based on ERIC PCR fingerprinting patterns using the Jacquard index and UPGMA clustering for P. aeruginosa isolates collected during Phase-1 (pre-intervention) from different hospital wards/units. The scale at the top represents percentage similarity. P. aeruginosa: Pseudomonas aeruginosa; ICU: intensive care unit; Med: medical; Onco: oncology; Ortho: orthopedics; Surg: surgery; Ped's: pediatrics; ERIC-PCR: enterobacterial repetitive intergenic consensus-polymerase chain reaction; UPGMA: unweighted pair-group method

Five major clusters were identified, but strains appear diverse in comparison with the *A. baumaunnii* isolates. Even within the adult ICU, a variety of strains appear to be circulating, with not much clonality being apparent. Three clusters were identified with *P. aeruginosa* isolates (Figure 4).

**Figure 5:**
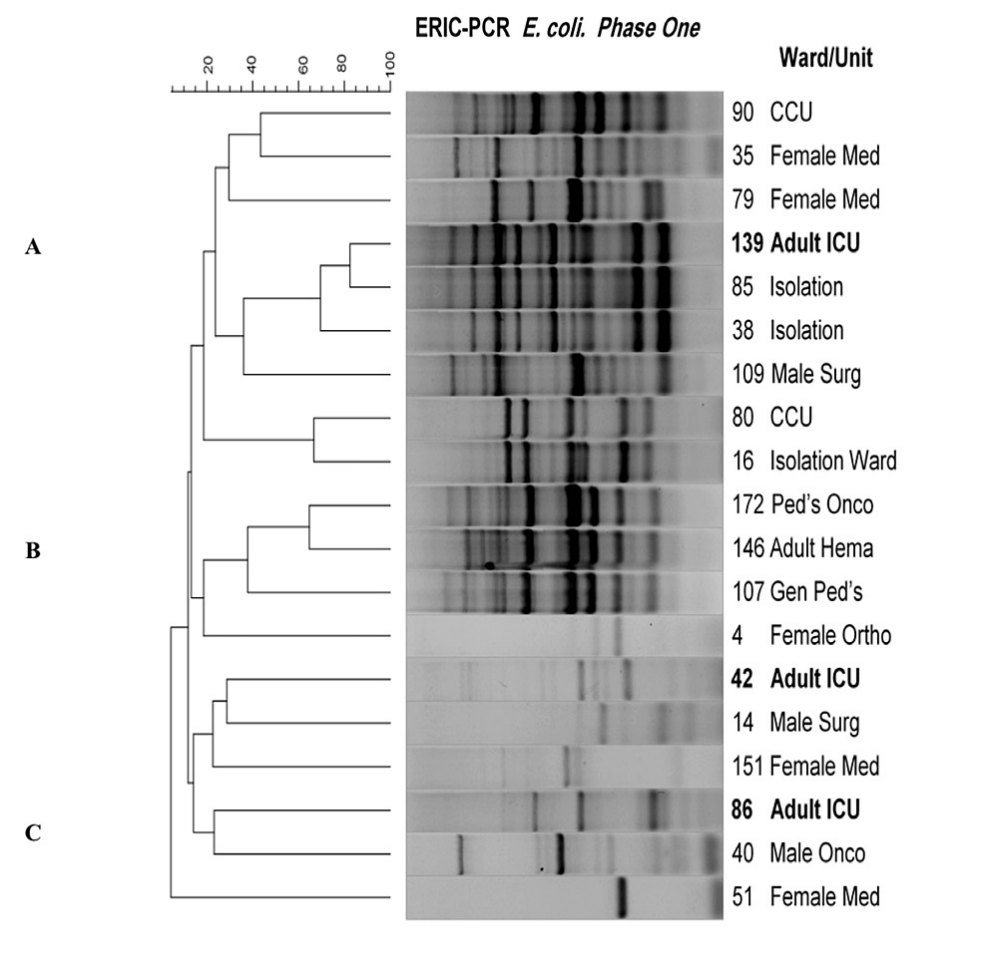
Dendrogram showing the cluster analysis based on ERIC PCR fingerprinting patterns using the Jacquard index and UPGMA clustering for E. coli isolates collected during Phase-1 (pre-intervention) from different hospital wards/units. The scale at the top represents percentage similarity. E. Coli: Escherichia coli; ICU: intensive care unit; CCU: cardiac care unit; Med: medical; Surg: surgery; Onco: oncology; Gen: general; Hema: hematology; Ped's: pediatrics; Ortho: orthopedics; ERIC-PCR: enterobacterial repetitive intergenic consensus-polymerase chain reaction; UPGMA: unweighted pair-group method

The adult ICU samples appear to be more similar (cluster B) compared to isolates from other sources and could have been transferred to male and female medical wards*. E. coli*, on the other hand, showed considerable diversity with respect to ERIC-PCR fingerprints (Figure 5).

It is interesting to note that sample 138 from the adult ICU shares a high degree of similarity (70 - 80%) to two isolation ward samples 85 and 38. Similarly, isolate 80 from the CCU and isolate 16 from the isolation ward also shared a high degree of similarity. A third group of interest is the similarity observed for strains 172, 146, and 107, which shared a high degree of similarity and were found in general pediatric, pediatric oncology, and adult hematology wards, respectively.

**Figure 6:**
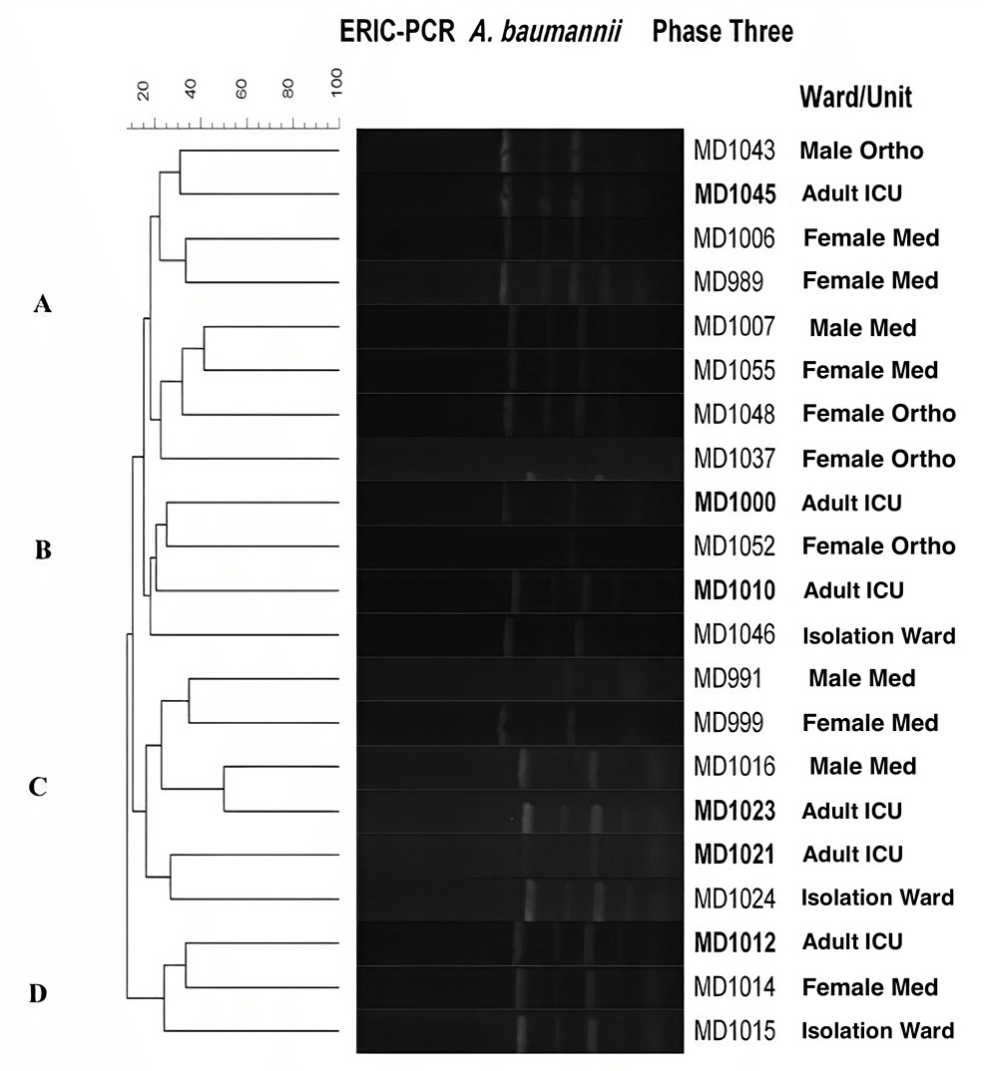
Dendrogram showing the cluster analysis based on ERIC PCR fingerprinting patterns using the Jacquard index and UPGMA clustering for A. Baumanii isolates collected during Phase-3 (post-intervention) from different hospital ward/units. The scale at the top represents percentage similarity. A. baumannii: Acinetobacter baumannii; ICU: intensive care unit; Med: medical; Surg: surgery; Ortho: orthopedics; ERIC-PCR: enterobacterial repetitive intergenic consensus-polymerase chain reaction; UPGMA: unweighted pair-group method

A total of 21 *A. baumannii *isolates were tested in Phase-3. More clonality was observed with the isolates, as was found in Phase-1. Four clusters were observed (Figure 6).

Adult ICU samples may have been transmitted to male and female orthopedic and medical wards since banding patterns display a high degree of similarity. Likely, the *A. baumannii *isolates observed in Phase-1 are still circulating in the adult ICU since fingerprints look highly similar.

## Discussion

*A. baumannii* is a significant cause of acute infections like bacteremia, ventilator-associated pneumonia, meningitis, and urinary tract infections among patients admitted to ICUs [14]. The results of ERIC-PCR carried out in Phase-1 of our study indicated that the* A. baumannii* isolates demonstrated a high degree of similarity, suggesting the transmission of the bacteria between patients within the adult ICU (Figure 2). The implementation of targeted interventions to prevent cross-transmission that started in Phase-2 of our study resulted in a 78% reduction in the rate of *A. baumannii* in the adult ICU. We repeated ERIC-PCR for *A. baumannii *isolated post-intervention only because of its significance as a result of ERIC-PCR done in Phase-1. Based on the presence of two predominant clonal types of *A. baumannii* post-intervention, it is helpful to mention that there is still a margin for improvement in infection control practice to eradicate it.

A study conducted by Cheon et al. (2016) in a South Korean hospital investigated the effectiveness of antimicrobial stewardship and comprehensive intensified infection control measures for controlling endemic MDR *A. baumannii *in ICUs [15]. The measures included restriction of carbapenem use, environmental cleaning, and disinfection in addition to basic infection control measures and contact precautions for patients who were colonized or infected with MDR *A. baumannii*. The incidence density rate of hospital-acquired MDR *A. baumannii* decreased from 22.82 cases per 1000 patient days to 2.68 cases per 1000 patient days after the implementation of the interventions [15]. Our study showed a significant reduction of *A. baumannii *after the implementation of infection control measures. The mean incidence of *A. baumannii* was reduced from 12.67 to 2.81 per 1000 patient days throughout the phases (from Phase 1 to Phase 3).

A similar study by Kochar et al. (2009) in one of the hospitals in New York also highlighted the importance of interventions. From 2004 to 2005, *Klebsiella pneumoniae carbapenemase (KPC)* producing *K. pneumoniae* was endemic in their ICU. Therefore, ICU was targeted for an enhanced infection control program. Multiple infection control measures were implemented, including all patients coming to their ICU with a culture positive for ceftazidime- or carbapenem-resistant gram-negative bacillus were positioned in contact isolation also, rectal swab samples were obtained from patients on admission to the ICU and weekly thereafter by means of culture swab. Additionally, improved decontamination of hands and environmental surfaces was applied in the ICU. The results post-interventions were comparable to our study in that they achieved a 62% reduction showing that intervention effectively decreased the incidence of *K. pneumoniae* in the ICU [16]. 

The diversity observed for *P. aeruginosa* indicated that it was less likely that the bacteria were being transmitted between patients due to the lack of infection prevention practices. These results suggest that a more effective antibiotic stewardship program and strict infection prevention measures are required to achieve the desired reduction in these particular bacteria. The results of a study conducted by Liu et al. (2018) in a Chinese hospital demonstrated that incidence rates of *P. aeruginosa* at a tertiary hospital decreased from 4.9 to 1.0 per 1000 patient days after implementing infection control interventions combined with an antibiotic stewardship program. The measures included environmental cleaning and disinfection, HH improvement, and education about infection control, together with antibiotic restriction through a classification management system for antibiotic use [17]. 

Compliance rates for HH in our study appear to be much higher than those observed in sub-Saharan Africa, with rates below 25% [18]. They are, however, much closer to the rates currently observed in developed countries which have now acquired a good culture of HH practices, according to WHO's 5 Moments [19]. It is almost not possible to evaluate the sole contribution of HH in lowering MDR-GNB. Perhaps HH must be implemented along with other measures/interventions (multimodal) to exert maximal effectiveness.

The interventions selected in our study did not include some of the interventions contained in recently published WHO guidelines for the prevention and control of carbapenem-resistant Enterobacteriaceae, *A. baumannii*, and *P. aeruginosa* in healthcare facilities, especially active screening of colonized patients because the active screen of colonized patients for MDR-GNB was not available in our institution [20]. Most of the other interventions in our study are recommended in WHO guidelines, except patients' baths with chlorhexidine solution. Many studies have proven chlorhexidine baths to help reduce the rates of MDR GNB infections [21,22]. The main limitation of the study is its single-center design. Another limitation was that it was conducted only for ICU patients.

## Conclusions

The IPC and stewardship interventions significantly reduced the incidence of common MDR-GNB in adult ICUs, albeit to ascertain the relative contributions of each. Such interventions must be conducted regularly to keep the incidence of MDR-GNB in check. Constant surveillance, proper antibiotics use, and implementation of adequate IPC measures are recommended to reduce the risk of MDR GNB infections.
